# Decellularized Extracellular Matrices and Cardiac Differentiation: Study on Human Amniotic Fluid-Stem Cells

**DOI:** 10.3390/ijms21176317

**Published:** 2020-08-31

**Authors:** Giulia Gaggi, Andrea Di Credico, Pascal Izzicupo, Silvia Sancilio, Michele Di Mauro, Giovanni Iannetti, Susanna Dolci, Giovanni Amabile, Angela Di Baldassarre, Barbara Ghinassi

**Affiliations:** 1 Haman Anatomy and Cell Differentiation Lab, Department of Medicine and Aging Sciences, University “G.d’Annunzio” of Chieti-Pescara, 66100 Chieti, Italy; giulia.gaggi@unich.it (G.G.); andrea.dicredico@unich.it (A.D.C.); izzicupo@unich.it (P.I.); silvia.sancilio@unich.it (S.S.); angela.dibaldassarre@unich.it (A.D.B.); 2 Cardio-Thoracic Surgery Unit, Heart and Vascular Centre, Maastricht University Medical Centre (MUMC), Cardiovascular Research Institute Maastricht (CARIM), 6202 Maastricht, The Netherlands; mdimauro1973@gmail.com; 3 University of Rome La Sapienza, 00185 Roma, Italy; iannetti98@gmail.com; 4 Department of Biomedicine and Prevention, University of Rome “Tor Vergata”, 00133 Rome, Italy; dolci@uniroma2.it; 5 Enthera Srl, 20123 Milan, Italy; giovanni.amabile@entherapharmaceuticals.com

**Keywords:** cardiac differentiation, cardiac tissue engineering, decellularized extracellular matrix, amniotic fluid stem cells, ^Cardiopoietic^AF cells, scaffolds, induced pluripotent stem cells, biomaterials, sarcomeric proteins, L-type calcium channels

## Abstract

Cell therapy with a variety of stem populations is increasingly being investigated as a promising regenerative strategy for cardiovascular (CV) diseases. Their combination with adequate scaffolds represents an improved therapeutic approach. Recently, several biomaterials were investigated as scaffolds for CV tissue repair, with decellularized extracellular matrices (dECMs) arousing increasing interest for cardiac tissue engineering applications. The aim of this study was to analyze whether dECMs support the cardiac differentiation of ^Cardiopoietic^AF stem cells. These perinatal stem cells, which can be easily isolated without ethical or safety limitations, display a high cardiac differentiative potential. Differentiation was previously achieved by culturing them on Matrigel, but this 3D scaffold is not transplantable. The identification of a new transplantable scaffold able to support ^Cardiopoietic^AF stem cell cardiac differentiation is pivotal prior to encouraging translation of in vitro studies in animal model preclinical investigations. Our data demonstrated that decellularized extracellular matrices already used in cardiac surgery (the porcine Cor^TM^PATCH and the equine MatrixPatch^TM^) can efficiently support the proliferation and cardiac differentiation of ^Cardiopoietic^AF stem cells and represent a useful cellular scaffold to be transplanted with stem cells in animal hosts.

## 1. Introduction

Cardiovascular (CV) diseases represent some of the main causes of mortality in worldwide [1], comprising 31% of global death [2,3,4]. The generation of fully differentiated cardiomyocytes (CMs) and cell-based tissue engineering approaches are among the goals of regenerative medicine. Cell therapy, with a variety of stem populations, is being increasingly investigated as a promising therapeutic approach to CV diseases [5,6].

The transplantation of beating CMs is expected to be more effective for serious heart diseases. The identification of the appropriate engineering processes and suitable culture substrates to induce stem cell differentiation into beating CMs is therefore pivotal [3]. Data obtained with human induced pluripotent stem cells (hiPSCs) showed that these cells can efficiently differentiate into patient-specific beating CMs in vitro [6,7,8], but their clinical use must be tempered due to their tumorigenic potential [5]. Recently, we proposed an alternative and safer stem cell source, demonstrating that human amniotic fluid cells that express the multipotency markers SSEA4, OCT4, and CD90 (^Cardiopoietic^AF cells), if properly cultured on Matrigel^®^ in presence of differentiative factors, can give rise to a morphologically homogeneous population of CMs that, similar to hiPSC-derived CMs, express cardiac-specific proteins and may contract spontaneously [2,9,10].

Nevertheless, even if ^Cardiopoietic^AF cells represent a promising source of CMs, the translation of their use in animal model preclinical studies is hampered by the need to identify an appropriate cellular scaffold. Indeed, despite its large use in “in vitro”, Matrigel^®^ cannot be used in “in vivo” human studies due to its murine origins and potential tumorigenicity [11]. The realization of an appropriate scaffold for the cardiac tissue engineering presents unique challenges, since native CMs are continuously exposed to cyclic mechanical (stress and strain) and electrical stimuli that must be properly transmitted to neighboring cells. In addition to these peculiar characteristics, tissue scaffolds for heart regeneration must also comply with the general requirements for all tissue scaffolds, such as degradation time, cell–scaffold interactions, vascularization/nutrient delivery, and implantation method [12,13]. Among the biological scaffolds, xenogenic, decellularized extracellular matrix (dECM) recently aroused a great deal of interest [3,14,15,16,17,18,19] with some matrices already being used in clinical practice; in fact, the elimination of cells from the tissues significantly reduces the risk of inflammatory response and immunological rejection, while preserving the natural three-dimensional structure of the ECM.

Cor^TM^ PATCH is a decellularized, porcine, small intestinal, submucosal extracellular matrix approved by the Federal Drug Administration to repair cardiovascular injury by providing a support for host cells to repair the damaged heart [20]. Evidence shows that the Cor^TM^ PATCH is resorbed in animal models, allowing the formation of nonfibrotic connective tissue without an accompanying inflammatory response [21,22,23].

The equine Matrix Patch^TM^ is a cell-free, equine-derived, pericardial patch routinely used in pediatric cardiac surgery, providing excellent mechanical properties, however, no calcification shrinkage has yet been observed [24].

The aim of this study was to evaluate dECM as a scaffold suitable for the adhesion, proliferation, and differentiation toward the cardiac lineage of ^Cardiopoietic^AF. For this reason, we analyzed the biological response of the ^Cardiopoietic^AF to two different dECM, the Cor^TM^ PATCH and the Matrix Patch^TM.^, which were obtained from different animal origins (porcine and equine, respectively). The abilities of ^Cardiopoietic^AF or hiPSCs (as a positive control) to adhere, proliferate, and differentiate in CMs when seeded onto the dECM were tested in comparison with the same cells cultured in Matrigel^®^, a substrate matrix known to support the process but which is not transplantable.

## 2. Results

We first investigated the capacity of seeding and the viability and differentiation potential toward the cardiac lineage of ^Cardiopoietic^AF on the porcine Cor^TM^ PATCH and the equine Matrix Patch^TM^. We performed the experiments in parallel using hiPSCs as a control of differentiation ability.

^Cardiopoietic^AF and hiPSC cells were expanded until 60% confluency and dissociated and replated on the dECMs prior to cardiac differentiation induction.

**Figure 1:**
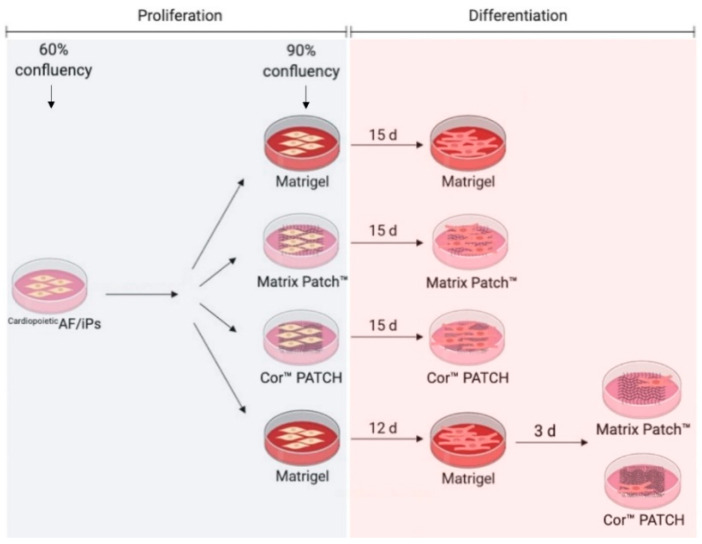
Scheme of the experimental design: (1) The ^Cardiopoietic^AF and the human induced pluripotent stem cells (hiPSCs) were differentiated in a monolayer for 15 days either on the Matrix Patch^TM^,the Cor^TM^ PATCH, or on the Matrigel^®^ control; (2) the ^Cardiopoietic^AF and the hiPSCs were differentiated in monolayer onto THE Matrigel^®^ for 12 days and then dissociated and moved to the Matrix Patch^TM^ or Cor^TM^ PATCH for 72 h.

Since we could not predict how the scaffolds would affect the viability or the differentiation potential of the cells, the evaluation was carried out via two parallel experimental procedures, which are summarized in Figure 1 and described as follows:

(1) The ^Cardiopoietic^AF and the hiPSCs were differentiated in a monolayer for 15 days, as previously described [2,25] either on one of the two dECMs or on the Matrigel^®^ control;
(2) The ^Cardiopoietic^AF and the hiPSCs were differentiated in a monolayer onto Matrigel^®^ for 12 days and then dissociated and moved to the dECMs for 72 h before analysis.

In the first experimental condition, both the undifferentiated hiPSCs and the ^Cardiopoietic^AF engrafted and proliferated easily on the dECMs and, when induced to differentiate, the viability was always comparable to the control cells differentiated on the Matrigel^®^.

Although a slight foreseeable drop in cell numbers was observed when the cells were cultured under cardiac permissive induction conditions (24 h of BMP4 and ActA followed by 72h of VEGF stimulation for mesodermal and cardiac induction, respectively), the cell vitality remained high (>82 ± 5% in hiPSCs and >76 ± 4% in ^Cardiopoietic^AF) after 15 days of differentiation in all experimental conditions.

**Figure 2:**
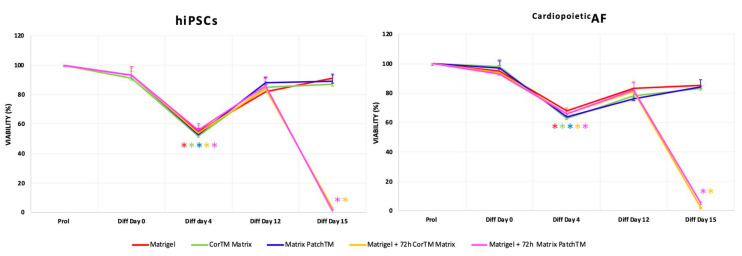
Percentage of hiPSCs and ^Cardiopoietic^AF viability, obtained by 3(4,5-Dimethylthiazol-2yl)-2,5-Diphenyl Tetrazolium Bromide (MTT) assay performed under proliferation conditions once cells reached 60% confluency and under differentiation conditions at day 0, 4, 12, and 15 in all experimental conditions, as indicated. The graphs show the mean ± SD of five independent experiments; * *p* < 0.05.

In contrast, the second experimental condition did not give the same encouraging results; only a small percentage of hiPSC- and ^Cardiopoietic^AF-derived CMs were able to attach to the dECMs once dissociated and no viable cells were present on the matrices after 72 h. Dissociated ^Cardiopoietic^AF-derived CMs were also unable to adhere and proliferate when reseeded on the Matrigel^®^ control (Figure 2). These observations suggest that both hiPSCs the ^Cardiopoietic^AF-derived CMs are deeply threatened by enzymatic dissociation, hampering their abilities to restore their cell–matrix and cell–cell interactions, thereby affecting their viability.

**Figure 3:**
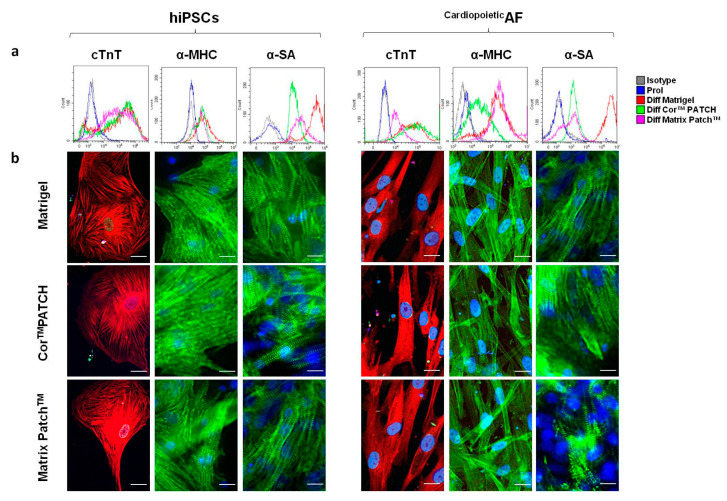
Detection of cTNT, α-MHC, and α-SA in hiPSC- and ^Cardiopoietic^AF-derived cardiomyocytes (CMs), obtained after 15 days of differentiation using Core^TM^ PATCH, Matrix Patch^TM^, and Matigel, as indicated. (**a**) Flow cytometry analysis of TnT, α-MHC, and α-SA in hIPSCs and ^Cardiopoietic^AF cells, as indicated. Legend of histogram colors: Grey: isotype control; blue: proliferating cells (undifferentiated); red: differentiation on Matrigel^®^; green: differentiation on Cor^TM^ Patch; purple: differentiation on Matrix Patch^TM^. (**b**) Immunofluorescent staining for cTnT (red), α-MHC (green), and α-SA (green) in hIPSCs and ^Cardiopoietic^AF cells on Core^TM^ PATCH, Matrix Patch^TM^, or Matrigel^®^, as indicated. The nuclei were counterstained with DAPI (blue). Scale bar: 10 μm. Data are representative of five independent experiments.

After 15 days of differentiation culture following the first experimental condition either on the dECMs or on the Matrigel^®^ internal control, we analyzed hiPSC- and ^Cardiopoietic^AF-derived CM expression of cTnT, α-MHC, and α-SA, specific sarcomeric proteins recognized as “late” cardiac markers. Sarcomeric proteins represent the structural building blocks of heart muscle, which are essential for contraction and relaxation [26]. Interestingly, differentiated cells obtained from both hiPSCs and ^Cardiopoietic^AF expressed high levels of cTnT, α-MHC, and α-SA, comparable in all the experimental conditions (Figure 3 and Table 1). In particular, the percentage and fluorescence intensity of the analyzed proteins were stackable for cTnT and a-MHC in hiPSCs and ^Cardiopoietic^AF cells cultured in the dECMs and Matrigel^®^, while α-SA of ^Cardiopoietic^AF cultured in the Matrix Patch^TM^ was less expressed, although still at a high percentage, than the same cells differentiated in Matrigel^®^. α-SA of the hiPSCs and ^Cardiopoietic^AF cells cultured in the Cor^TM^ PATCH remaiedn comparable to those differentiated in Matrigel^®^, although slightly less expressed (Table 1 and Figure 3). Nevertheless, immunofluorescence images displayed clearly that the sarcomeric striations typical of α-SA and cTnT localization were present in all culture conditions, indicating that both dECMs are permissive to cardiac differentiation (Table 1 and Figure 3).

**Figure 4:**
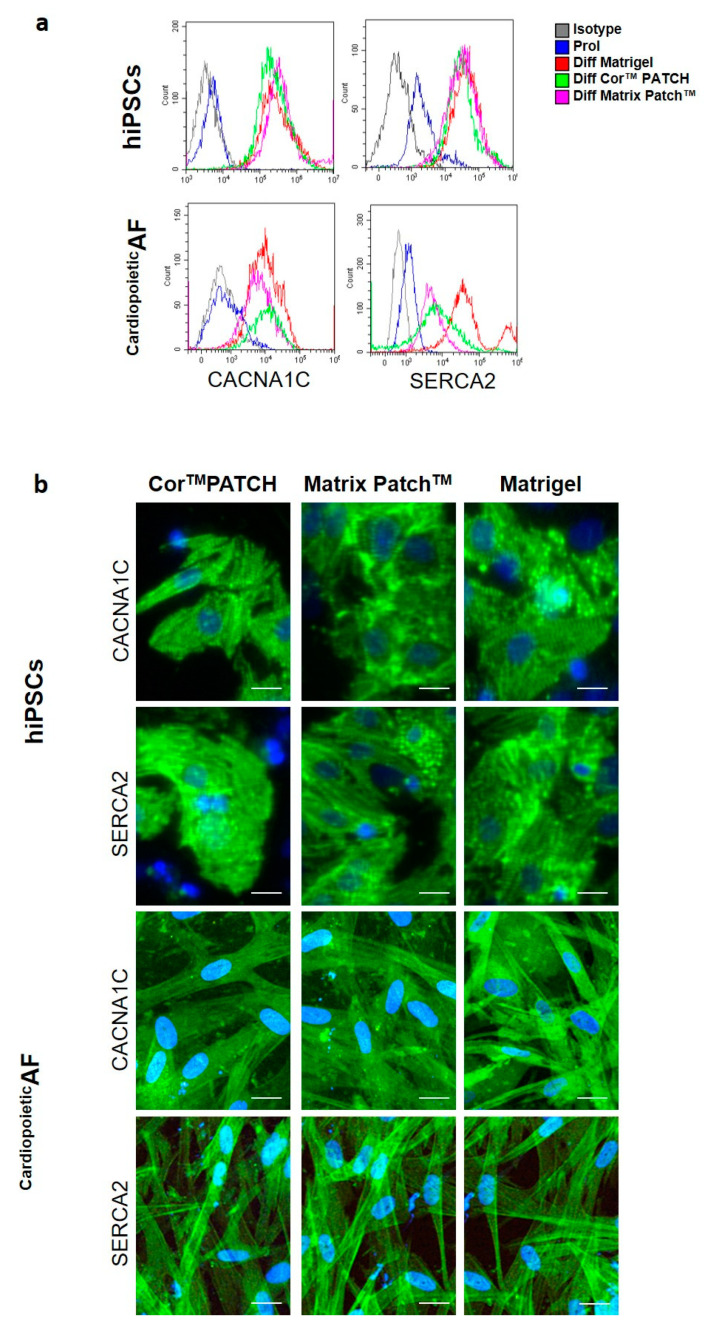
Detection of calcium pumps CACNA1C and SERCA2 in hiPSC- and ^Cardiopoietic^AF-derived CMs obtained after 15 days of differentiation. (**a**) Flow cytometry analysis of CACNA1C and SERCA2 on hIPSCs and ^Cardiopoietic^AF cells, as indicated. Legend of histogram colors: Grey: isotype control; blue: proliferating cells; red: differentiation on Matrigel^®^; green: differentiation on Cor^TM^ Patch; purple: differentiation on Matrix Patch^TM^. (**b**) Immunofluorescent staining for CACNA1C and SERCA2 (green) in hiPSCs and ^Cardiopoietic^AF cells, as indicated. Nuclei were counterstained with DAPI (blue). Scale bar: 10 μm. Data are representative of five independent experiments.

Flow cytometry and immunofluorescence revealed the dramatic induction of the L-type calcium channel CACNA1C and SERCA2 proteins, two calcium pumps essential for excitation–contraction coupling that reside in the sarcolemma and sarcoplasmic reticulum of CMs, respectively (Table 1 and Figure 4a). In the hiPSCs and ^Cardiopoietic^AF cells differentiated in the dECMs and in Matrigel^®^, CACNA1C was localized mainly in the perinuclear area and marked the cytoplasmic membrane in some cells, probably indicating a homing of the protein in the sarcolemma of more mature CMs. On the other hand, SERCA2 immunolabeling showed the typical reticulated organization of the sarcoplasmic reticulum (Figure 4b).

**Figure 5:**
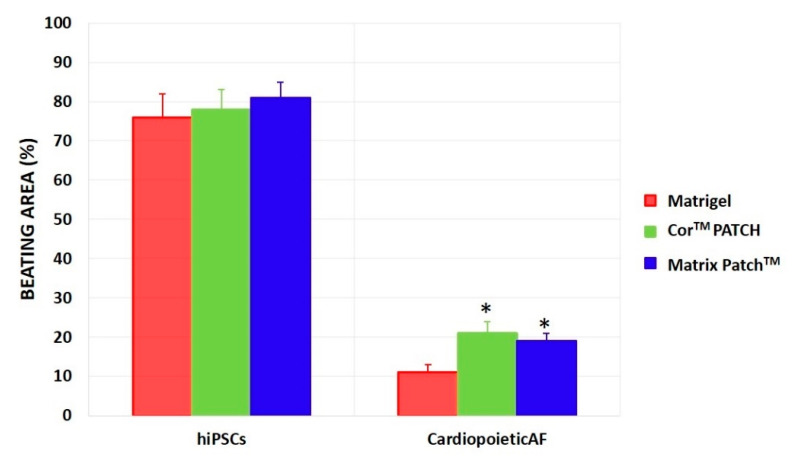
Percentage of dish area of hiPSC- and ^Cardiopoietic^AF-derived CMs that presented spontaneous beating foci after 15 days of differentiation in Matrigel^®^ (red), Cor^TM^ PATCH (green), or Matrix Patch^TM^ (blue). hiPSC-derived CMs were lower compared to ^Cardiopoietic^AF-derived CMs differentiated on the Cor^TM^ PATCH and the Matrix Patch^TM,^ compared to Matrigel^®^. The graphs show the mean ± SD of five independent experiments; * indicates statistically differences (*p* < 0.05) of the Cor^TM^ PATCH or the Matrix Patch^TM^ culture vs. the same cells cultured in Matrigel^®^.

Finally, the beating colonies appeared from 8 days of culture and increased until 15 days on almost all of the substrates. hiPSC- and ^Cardiopoietic^AF-derived CMs differentiated in both dECMs showed percentages of spontaneous beating foci comparable to or even higher than the same cells differentiated in Matrigel (Figure 5), demonstrating once again that both the Cor^TM^ PATCH and Matrix Patch^TM^ could be considered suitable scaffolds for the adhesion and the proliferation of stem cells from different sources and permissive for differentiation toward cardiac lineage.

## 3. Discussion

The ideal goal of stem cell therapy is to substitute necrotic or dysfunctional cardiac tissue with new competent CMs derived from stem cells [27,28]. Currently, the only cells with proven promising results regarding cardiogenic potential are hiPSCs. The possibility to obtain functionally mature CMs from patient-specific hiPSCs is a big challenge for clinical use in tailored cell therapy [5,29,30,31,32,33], with their epigenetic instability still considered an unsolved issue [10]. The recent discovery that ^Cardiopoietic^AF could circumvent any ethical and safety concerns and provide effective cell replacement therapy for cardiac diseases, particularly in congenital heart defect repair applications, [2,34,35] opened the door to a new scenario.

For the first time to our knowledge, ^Cardiopoietic^AF (a new and safe source of stem cells) were demonstrated to be able to proliferate on dECMs and generate CMs with high efficiency in this work. In line with the literature [7,36] cardiac differentiation from hiPSCs and ^Cardiopoietic^AF cells was observed to be optimal in terms of both efficacy and efficiency when cultured in a monolayer on Matrigel^®^, a 3D matrix known to support cells in “in vitro” studies, but is unfortunately not transplantable for safety reasons [37]. In this study, we replaced the Matrigel^®^ with dECMs and investigated the biological response of ^Cardiopoietic^AF cells to two engineered patches, the pericardial Cor^TM^ PATCH and the Matrix Patch^TM^. Both technologies are decellularized membranes, one derived from porcine intestinal submucosa, the other from equine pericardium, which are commonly used in clinical practice, mainly as patches to restore valves and vascular tissue in need of repair; when resorbed, they allow the formation of nonfibrotic connective tissue. Neither were previously analyzed as possible transplantable scaffolds for CMs generated “in vitro”.

dECMs were widely explored as natural scaffolds for cardiac tissue engineering applications because they offer many unique advantages, such as preservation of organ-specific ECM microstructure and composition, retention of tissue-mimetic mechanical properties, and biochemical cues in favor of subsequent recellularization [12,38,39]. In this study, we exploited two dECMs from small intestinal submucosa and bovine-derived pericardium that were previously successfully tested in clinical studies [40] dECM scaffolds can be implanted during cardiac surgery intervention onto injured myocardial regions since they offer a structural support to the damaged myocardium and stimulate endogenous mechanisms of tissue repair, such as vasculogenesis [40]. Our data suggest their potential use as biological scaffolds to support ^Cardiopoietic^AF cell proliferation and cardiac differentiation, with the ideal goal of implantation at the site of the infarction to restore tissue function. Interestingly, dECMs derived from both cardiac and intestinal tissues showed similar results, with no substantial differences in cellular adhesion or differentiation efficiency.

Another important observation was that both hiPSCs and ^Cardiopoietic^AF needed to be cultured from the beginning on the dECMs (first experimental condition of this manuscript) in order to guarantee proper engrafting and to maintain their differentiation features. This was unsurprising, as previous studies showed that most of the murine embryonic stem cell (mESC)-derived CMs failed to attach to a variety of polymer substrates at the end of the differentiation process. It is therefore probable that CMs need a more complex environment than 2D or 3D scaffolds enriched in cardioprotective factors to attach and survive after dissociation [41]. In addition, Van Deel et al. showed that the commonly used enzymatic detaching methods, such as Trypsin, Detachin, and Accutase, are lethal to adult CMs, altering the integrity of the cells [42]. Thus, the failure of CMs obtained from hiPSCs and ^Cardiopoietic^AF cells to attach onto dECMs was probably due to the fact that, after enzymatic digestion, the CMs were not able to recreate cell–cell and cell–scaffold connections. However, further studies are necessary to address this point.

^Cardiopoietic^AF stem cells attached to and proliferated easily on the two engineered scaffolds, and, if induced to differentiate, gave rise to CMs that showed typical sarcomere striation, expressed mature CMs hallmark proteins (α-AS, α-MHC, and cTnT) [5,43] and showed strong CACNA1 and SERCA2 expression, two L-type calcium channels essential for the so-called “Ca^2+^-induced Ca^2+^ release”, the mechanism crucial for excitation–contraction coupling in mammalian cardiac muscle. ^Cardiopoietic^AF-derived CMs cultured on dECMs showed a proper homogeneous cardiac phenotype [43] and expressed cardiac-specific functional markers comparable to those obtained using the Matrigel^®^ culture.

Finally, hiPSC-derived CMs differentiated using both dECMs showed percentages of spontaneous beating foci comparable to the same cells differentiated using Matrigel^®^, while the percentage of the contracting area in ^Cardiopoietic^AF-derived CMs was surprisingly significantly higher in both the Cor^TM^ PATCH and Matrix Patch^TM^ than in the control cells. Even if this percentage never exceeded 30% of the total area, it was still a promising result, since very few groups were able to obtain beating CMs from amniotic stem cells without prior reprogramming [2,44,45]. This was possibly because, as expected [5,46,47] CMs with different levels of maturity were obtained, and a scaffold with a rigid elasticity niche may strongly promote beating induction, in line with what was reported by Hirata and colleagues [48].

In conclusion, these data confirm the efficacy of both the analyzed dECMs as cell carriers for ensuring phenotypic and functional ^Cardiopoietic^AF-derived CMs, driving new challenges for producing biomimetic cardiac patches in tailored regenerative medicine.

The main limitation of this study was the lack of specific analysis of the calcium-handling capabilities of ^Cardiopoietic^AF-derived CMs differentiated using the Cor^TM^ PATCH and the Matrix Patch^TM^. More efforts are needed to characterize the electrophysiological properties and to promote of the homogeneity of ^Cardiopoietic^AF-derived CMs prior to encouraging their transplantation in animal models.

## 4. Materials and Methods

### 4.1. Isolation and Culture of CardiopoieticAF and hiPSC Cells

AF samples were obtained from 8 women undergoing amniocentesis for prenatal diagnosis at 16–17 weeks of pregnancy after written informed consent, in accordance with the Declaration of Helsinki. All samples presented normal diploid male karyotypes, as evidenced by cytogenetic investigation. The mean (±SD) maternal age at amniocentesis was 37.9 ± 2.6 years. The study was approved by the local ethics committee and all experiments were performed in accordance with relevant guidelines and regulations.

^Cardiopoietic^AF cells were isolated as previously described on the basis of the phenotypic pattern [2] cultured in Iscove’s modified Dulbecco’s medium (IMDM, Lonza Group Ltd., Basel, Switzerland), supplemented with 20% fetal bovine serum (FBS, GE Healthcare HyClone Defined Fetal Bovine Serum, Buckinghamshire, UK), 100 U/mL penicillin, 100 µg/mL streptomycin, 2 mM L-glutamine, (all Sigma-Aldrich, Saint Louis, MO, USA), and 5 ng/mL basic fibroblast growth factor (bFGF, Invitrogen, Thermo Fisher Scientific, Waltham, MA, USA), and incubated at 37 °C with 5% humidified CO_2_.

hiPSCs [49] were cultured on a monolayer of irradiated murine fibroblasts (Thermo Fischer Scientific, Waltham, MA, USA) in Dulbecco’s Modified Eagle’s Medium/Nutrient Mixture F-12 (DMEM/F12) supplemented with 20% KnockOut Serum Replacement (KO) (Thermo Fischer Scientific, Waltham, MA, USA), 1% penicillin/streptomycin (Thermo Fischer Scientific, Waltham, MA, USA), 2 mM L-glutamine (Corning, New York, NY, USA), 5 ng/mL basic fibroblast growth factor (bFGF) (Thermo Fischer Scientific, Waltham, MA, USA), and 1% MEM nonessential amino acid (Corning, New York, NY, USA).

### 4.2. Culture in Monolayer and Cardiac Differentiation

^Cardiopoietic^AF and hiPSCs were seeded either onto Matrigel^®^ (Corning, Flintshire, UK)-coated plates or onto two dECMs ((Cor^TM^ PATCH (CorMatrix, Roswell, GA, USA) or Matrix Patch^TM^ (Auto Tissue Berlin GmbH)) using two methods: a) The cells were seeded onto the scaffold before starting the differentiation protocol and were kept on the dECMs for the duration of the process, followed by analysis; b) the cells were differentiated onto Matrigel^®^ and after 12 days of differentiation were detached using 0.025% Tryspin-EDTA (Invitrogen Carlsband, CA, USA) and distributed on each dECM or back to the Matrigel^®^ control. Sample were cultured in differentiation conditions for 72 h and subsequently analyzed. The cells were cultured onto the dECM as described above, then, when 80–90% confluence was reached, the medium was changed to RPMI 1640 (Thermo Fischer Scientific, Waltham, MA, USA) supplemented with B-27 minus insulin (GIBCO, Thermo Fischer Scientific, Waltham, MA, USA), 50 μg/mL ascorbic acid (Sigma-Aldrich, Saint Louis, MO, USA) and 10 μM 5-aza-2′-deoxycytidine (5-Aza), and differentiation was induced by exposure for 24 h to activin A (50 ng/mL, R&D Systems, Minneapolis, MN, USA) and BMP4 (25 ng/mL, R&D Systems, Minneapolis, MN, USA), followed by treatment for 72 h with VEGF (10 ng/mL, R&D Systems, Minneapolis, MN, USA). Cells were analyzed after 15 days of differentiation. Beating foci were observed and quantified microscopically as the percentage of the beating area inside the whole plate [50,51].

### 4.3. MTT(3(4,5-Dimethylthiazol-2yl)-2,5-Diphenyl Tetrazolium Bromide) Assay

Cell viability and metabolic activity were measured using an MTT (3(4,5-dimethylthiazol-2yl)-2,5-diphenyl tetrazolium bromide) growth assay (Sigma-Aldrich, Saint Louis, MO, USA), as previously described [52]. The cultured medium was removed and washed once with 1X PBS. MTT solution (0.5 mg/mL; Sigma-Aldrich, Saint Louis, MO, USA) was added into each well and incubated for 4 h at 37 °C for formazan crystal development. Formazan crystals were dissolved in dimethyl sulfoxide (DMSO) (Sigma-Aldrich, Saint Louis, MO, USA) and read at 540 nm in a microplate reader.

The values obtained in the absence of cells were considered to be background and subtracted from the optical density values of the samples. Five independent experiments were performed under the same experimental conditions.

### 4.4. Flow Cytometry and Imaging Flow Cytometry

For flow cytometry and imaging flow cytometry, cells were treated with the FIX and PERM^®^ Kit (Thermo Fischer Scientific, Waltham, MA, USA) and incubated for 1 h at RT with anti-α-myosin heavy chain (α-MHC; 1:100; Abcam, Cambridge, UK), anti-cardiac troponin T (cTnT, 1:100; Abcam, Cambridge, UK), anti-α-sarcomeric actin (α-SA; 1:100; Abcam, Cambridge, UK), anti-sarcolemmal Ca^2+^ channel (CACNA1C, 1:100; Abcam, Cambridge, UK), and anti-sarcoendoplasmic reticulum Ca^2+^ ATPase protein (Serca2, 1:100; Invitrogen, Thermo Fisher Scientific, Waltham, MA, USA), followed by incubation with the appropriate secondary antibody conjugated with Alexa Fluor 488, PE-Alexa Fluor 647, or PE-Cy7 (1:200; Invitrogen, Thermo Fisher Scientific, Waltham, MA, USA), as previously reported [53]. Cells incubated with isotypes (all from BD) were used as negative controls [54].

Cytometric analysis was performed with a Cytoflex cytometer (Beckman Coulter, Brea, CA, USA) and data were analyzed using FlowJo (TreeStar, Ashland, OR, USA) or CytExpert Acquisition and Analysis Software (Beckman Coulter, Brea, CA, USA).

### 4.5. Immunofluorescence Staining and Microscopy

Cells fixed with 4% paraformaldehyde and permeabilized with 0.5% Triton X-100 were incubated with anti-cTnT (1:100, Abcam, Cambridge, UK), anti-α-MHC (1:100, Abcam, Cambridge, UK)), anti-α-SA (1:100, Abcam, Cambridge, UK), anti-CACNA1C (1:100, Abcam, Cambridge, UK), and anti-SERCA2 (1:100; Invitrogen, Thermo Fisher Scientific, Waltham, MA, USA), followed by the appropriate secondary antibody conjugated with Alexa Fluor 488 or PE (1:200; Invitrogen, Thermo Fisher Scientific, Waltham, MA, USA), as previously reported [51]. Nuclei were counterstained with DAPI (Invitrogen, Thermo Fisher Scientific, Waltham, MA, USA). Samples treated only with the secondary antibody were used as controls. Images were acquired using an Axio Vert A1 microscope. Images were analyzed by ZEN 2009 (Carl Zeiss, Oberkochen, Germany).

### 4.6. Statistical Analysis

All quantitative data were presented as the mean ± SD. Statistical comparisons were performed using one-way analysis of variance (ANOVA) and Student’s t-test [55]. The level of significance was set at *p* < 0.05.

## Figures and Tables

**Table 1:** Percentage of cells positive for cTnT, α-MHC, α-SA, CACNA1C, and SERCA2 in hiPSC- and ^Cardiopoietic^AF-derived CMs after 15 days of differentiation.

	cTnT	α-MHC	α-SA	CACNA1C	SERCA2
**hiPSCs**					
Prol	1.0 ± 0.5	1.2 ± 0.5	1.1 ± 1.1	6.5 ± 5.8	26.6 ± 9.5
Diff Matrigel^®^	83.5 ± 6.6 *	83.4 ± 3.1 *	95.3 ± 3.1 *	79.9 ± 6.8 *	93.4 ± 8.6 *
Diff Cor^TM^ PATCH	88.1 ± 11.1 *	69.2 ± 9.9 *	88.5 ± 7.3 *	76.2 ± 3.1 *	83.6 ± 7.3 *
Diff Matrix Patch^TM^	86.2 ± 5.6 *	81.4 ± 7.5 *	81.5 ± 6.4 *^&^	75.9 ± 6.2 *	86.1 ± 7.2 *
**^Cardiopoietic^AF**					
Prol	2.2 ± 2.1	1.1 ± 0.5	4.1 ± 2.1	12.5 ± 8.8	45.1 ± 12.5
Diff Matrigel^®^	85.5 ± 5.6 *	88.4 ± 8.1 *	89.3 ± 7.9 *	88.9 ± 5.9 *	83.5 ± 6.8 *
Diff Cor^TM^ PATCH	78.6 ± 9.6 *	79.5 ± 6.9 *	78.5 ± 7.4 *	96.2 ± 4.1 *	84.5 ± 9.3 *
Diff Matrix Patch^TM^	75.2 ± 6.6 *	74.4 ± 8.8 *	66.5 ± 9.4 *^&^	92.4 ± 7.2 *	83.5 ± 6.1 *

* *p* < 0.01 relative to cells under proliferation conditions (Prol); ^&^ *p* < 0.05 relative to cells differentiated in Matrigel (Diff Matrigel).
